# Meta-Analysis of Pulsed-Field Gel Electrophoresis Fingerprints Based on a Constructed *Salmonella* Database

**DOI:** 10.1371/journal.pone.0059224

**Published:** 2013-03-14

**Authors:** Wen Zou, Hung-Chia Chen, Kelley B. Hise, Hailin Tang, Steven L. Foley, Joe Meehan, Wei-Jiun Lin, Rajesh Nayak, Joshua Xu, Hong Fang, James J. Chen

**Affiliations:** 1 Division of Bioinformatics and Biostatistics, National Center for Toxicological Research, U.S. Food and Drug Administration, Jefferson, Arkansas, United States of America; 2 Granduate Institute of Biostatistics and Biostatistics Center, China Medical University, Taichung, Taiwan; 3 PulseNet Database Unit, Enteric Diseases Laboratory Branch, Division of Foodborne, Waterborne, and Environmental Diseases, National Center for Emerging and Zoonotic Infectious Diseases, Centers for Disease Control and Prevention (CDC), Atlanta, Georgia, United States of America; 4 Division of Microbiology, National Center for Toxicological Research, U.S. Food and Drug Administration, Jefferson, Arkansas, United States of America; 5 Department of Applied Mathematics, Feng Chia University, Taichung, Taiwan; 6 The Office of Scientific Coordination, National Center for Toxicological Research, U.S. Food and Drug Administration, Jefferson, Arkansas, United States of America; Belgian Nuclear Research Centre SCK/CEN, Belgium

## Abstract

A database was constructed consisting of 45,923 *Salmonella* pulsed-field gel electrophoresis (PFGE) patterns. The patterns, randomly selected from all submissions to CDC PulseNet during 2005 to 2010, included the 20 most frequent serotypes and 12 less frequent serotypes. Meta-analysis was applied to all of the PFGE patterns in the database. In the range of 20 to 1100 kb, serotype Enteritidis averaged the fewest bands at 12 bands and Paratyphi A the most with 19, with most serotypes in the 13−15 range among the 32 serptypes. The 10 most frequent bands for each of the 32 serotypes were sorted and distinguished, and the results were in concordance with those from distance matrix and two-way hierarchical cluster analyses of the patterns in the database. The hierarchical cluster analysis divided the 32 serotypes into three major groups according to dissimilarity measures, and revealed for the first time the similarities among the PFGE patterns of serotype Saintpaul to serotypes Typhimurium, Typhimurium var. 5-, and I 4,[5],12:i:-; of serotype Hadar to serotype Infantis; and of serotype Muenchen to serotype Newport. The results of the meta-analysis indicated that the pattern similarities/dissimilarities determined the serotype discrimination of PFGE method, and that the possible PFGE markers may have utility for serotype identification. The presence of distinct, serotype specific patterns may provide useful information to aid in the distribution of serotypes in the population and potentially reduce the need for laborious analyses, such as traditional serotyping.

## Introduction

Foodborne diseases are an important public health burden in the United States. In 2011 the Centers for Disease Control and Prevention (CDC) estimated that each year roughly 1 in 6 Americans (or 48 million people) gets sick, 128,000 are hospitalized, and 3,000 die due to foodborne illnesses, and nontyphoidal *Salmonella* is one of the leading causes among the 31 known foodborne pathogens [1]. The incidence of *Salmonella* infections has changed considerably over time, including changes in the frequency of antimicrobial-resistant *Salmonella* subtypes and the frequency of different serotypes among isolates associated with human infections [2]. Of the 2,541 *Salmonella* serotypes described as of 2007, 1531 were classified as serotypes of *Salmonella enterica* subsp. *enterica*, which causes more than 99% of *Salmonella* infections in humans [2]. Contaminated foods have been identified as the primary sources of human *Salmonella* infections [1]. To efficiently detect and prevent human salmonellosis, the development of rapid and sensitive *Salmonella* subtyping methods is of significant importance.

Multiple phenotypic and genotypic methods have been developed for *Salmonella* subtyping [3]. Traditional serotyping, which is based on the Kauffmann-White Scheme [4], has served as the basis for *Salmonella* serotype differentiation [5]. Pulsed field gel electrophoresis (PFGE) was adopted for national *Salmonella* surveillance and outbreak research in the 1990s, and has been successfully used in typing *Salmonella* from human patients, foods, and food animal sources because of its remarkable discriminatory power and high reproducibility [3], [6]. Amplified fragment length polymorphisms (AFLP) is based on the selective amplification of genomic restriction fragments by PCR, and has been successfully used in bacterial taxonomy and typing schemes for the differentiation of highly related pathogen bacterial strains [7]−[11]. Additionally, DNA sequence-based subtyping methods, including DNA microarray analysis [12], [13], multi-locus sequence typing (MLST) [14], [15], multi-locus variable-number tandem repeat analysis (MLVA) [16], [17] have been applied to the identification and tracking of salmonellosis outbreaks [3]. Recently, next-generation sequencing (NGS) has begun to be applied in *Salmonella* outbreak strain identification and source tracking [18]−[22]. This approach is a powerful method for differentiating highly clonal outbreak strains [18].

All the subtyping approaches have their own strengths and weaknesses in terms of sensitivity, cost, speed, and robustness [3], [23], [24]. Although PFGE is considered as time-consuming, labor intensive, and provides less-detailed genetic information than NGS methods [23], it is currently the most widely used molecular subtyping method for *Salmonella* [25] and is routinely used in CDC and state health labs in the United States. PulseNet (http://www.cdc.gov/pulsenet), the CDC coordinated molecular surveillance network used for foodborne infection in the United States, has the largest and most valuable *Salmonella* database in the world. It has collected more than 350,000 PFGE patterns, including outbreak strains covering more than 500 serotypes, since 1996 [26]. Since PulseNet has set up a standard protocol for obtaining and processing the gel images, PFGE fingerprints from various laboratories are reproducible and comparable [26]. The valuable data in PulseNet provide the opportunity to study the global ecology, epidemiology, transmission, and evolution of the emerging *Salmonella* serotypes from PFGE profiles.

In this study, we have surveyed the data in PulseNet and created a database of PFGE patterns of the most frequent serotypes isolated from human sources. The constructed PFGE database was stored in the Intranet of the US Food and Drug Administration's (US/FDA) National Center for Toxicological Research. The primary objective of this study is to present a meta-analysis of this large database to systematically investigate and characterize the phylogenetic relationships between PFGE patterns of *Salmonella* serotypes. For each of the 32 most frequent serotypes associated with outbreaks, we proposed that there would be predominant bands or band combinations that when examined using meta-analysis of a large data set would be useful as predictive markers for the particular serotypes. We investigated the diversity of PFGE patterns within each serotype and distinguished the relationships between various *Salmonella* serotypes. The results provide a better understanding of *Salmonella* genetic diversity and epidemiology, and can help in the application of PFGE-based characterization and surveillance of *Salmonella* isolates in outbreak source tracking.

## Materials and Methods

### Database construction of PFGE fingerprints

A total of 45,923 *Xba*I-PFGE patterns of *Salmonella enterica* isolates were collected for the database (Table 1). These patterns were randomly selected to include each of the 32 most frequent serotypes from all the submissions from human sources to PulseNet from 2005 to 2010. More than 99% of the isolates were collected from stool, blood, urine or unknown sites of human sources in the US. Less than 1% of the isolates came from other countries.

**Table 1:** The composition of the database of *Salmonella* PFGE fingerprints.

Serotypes	Number of patterns	Ranks*	Total/1996−2009*	Percent/1996−2009*
Agona	1954	14	7376	1.4
Braenderup	2008	13	7807	1.5
Enteritidis	2338	2	90328	17.4
Hadar	1981	19	5263	1.0
Heidelberg	2114	4	24819	4.8
I 4,[5],12:i:-	2281	11	7912	1.5
Infantis	2078	12	7857	1.5
Javiana	2102	5	19170	3.7
Mississippi	1999	16	5430	1.0
Montevideo	2041	6	12855	2.5
Muenchen	1970	8	10652	2.1
Newport	2005	3	44483	8.6
Oranienburg	1951	10	9042	1.7
Paratyphi B var. L(+) tartrate+	2011	18	5305	1.0
Poona	1956	20	4101	0.8
Saintpaul	2252	9	9606	1.9
Thompson	2045	15	7208	1.4
Typhi	1941	17	5371	1.0
Typhimurium	2064	1	91028	17.6
Typhimurium var. 5-	2146	7	12688	2.4
		Sub Total	388301	74.9
Anatum	478	23	2863	0.6
Bareilly	426	24	2829	0.5
Berta	502	21	3059	0.6
Derby	393	30	2080	0.4
Hartford	531	27	2431	0.5
Litchfield	401	28	2424	0.5
Mbandaka	432	25	2727	0.5
Panama	516	29	2206	0.4
Paratyphi A	135	35	1678	0.3
Schwarzengrund	225	26	2544	0.5
Senftenberg	189	32	1989	0.4
Stanley	460	22	2914	0.6
		Sub Total	418045	80.6
Total	45923	Total/1996−2009	518419	100.0

* : The table shows the information on *Salmonella* isolates from human sources during 1996−2009, which was derived and calculated from the *Salmonella* Annual Summary of 2006

(http://www.cdc.gov/ncidod/dbmd/phlisdata/salmtab/2006/SalmonellaTable1_2006.pdf) and the *Salmonella* Annual Summary Tables 2009 (http://www.cdc.gov/ncezid/dfwed/PDFs/SalmonellaAnnualSummaryTables2009.pdf)

To store the patterns in the database, the gel images were processed and analyzed by BioNumerics software (Applied Maths, Inc., Austin, TX, Version 6.0) according to the PulseNet protocol [27]. The band matching was performed at a trace-to-trace optimization value of 1.56% and a band position tolerance set at 1%. Because the BioNumerics software can process a maximum of 20,000 PFGE patterns simultaneously, the data were randomly split into three groups. Since the band classes for the three groups were created separately, a standardization procedure was needed before the combination of the three groups. Two methods, the BioNumerics fixed band method and NCTR fixed band method, were developed to standardize the band classes for cross-group analysis [28]. Subsequent to the normalization procedure, the three groups were combined.

### Characterization of patterns and serotypes of the database

The normalized band matching for 45,923 PFGE patterns was exhibited in a single Excel file with band presence or absence at each band location coded as 1 or 0, respectively. The band number for each pattern and the mean band number of each serotype, between 20 to 1100 kb were calculated. For each serotype, the proportion of band occurrences at each designated location was measured, and the 10 most frequent bands were sorted by frequency.

### Distance Matrix Development

To identify the differences and relationships among the various *Salmonella* patterns and among the 32 serotypes in the database, the distance matrix for 32 serotypes was computed. The normalized database consisting of 45,923 patterns from 32 serotypes was applied. The distance matrix presented the dissimilarities for any two patterns in the entire database. The dissimilarity of PFGE patterns inter- or intra-serotypes was calculated by Jaccard Distance [29], and the values ranged from 0 (green) to 1 (red).

### Hierarchical cluster analysis

The characteristic parameters of each serotype were obtained by calculating the proportions of the bands present at every designated band location with values ranging from 0 to 1. The hierarchical cluster analysis was applied based on the dissimilarity measures of any two serotypes calculated by the Euclidean distance [30] of the characteristic parameters. In this study, two-way clustering analysis was applied, in which both serotypes and band locations were clustered according to dissimilarity measures to identify the associations between serotypes and band locations simultaneously.

## Results

### Construction of the database of PFGE fingerprints

Based on the statistics of the *Salmonella* Annual Report 2006 [2] and *Salmonella* Annual Summary Tables 2009 from CDC [31], we calculated the frequencies of the serotypes and decided to include the 20 most frequently occurring serotypes and another 12 serotypes ranking between the top 21 and the top 35 in our database (Table 1). The three right hand columns in Table 1 list the total numbers of patterns, ranks and percentages of the 32 serotypes over 14 years, from 1996 to 2009 [2], [31]. All together, the isolates of the 32 serotypes comprised 80.6% of all isolates reported within 14 years nationwide, within which the 20 most common serotypes covered 74.9% and the 12 less common serotypes only 5.7%.

To meet the differences of the occurring frequencies of the 32 serotypes, we randomly selected approximately 2000 PFGE patterns for each of the 20 most frequent serotypes, and between 400 to 500 patterns for each of the 12 less frequent serotypes (with the exception of serovars Paratyphi A, Schwarzengrund, and Senftenberg due to shortage of pattern data) from PulseNet. The entire database consisted of 45,923 randomly selected PFGE patterns for the 32 *Salmonella* serotypes. We assumed that our database constructed in this study had similar coverage and representation of *Salmonella* serotypes and patterns occurred from 1996 to 2009.

### Database Characterization

**Figure 1:**
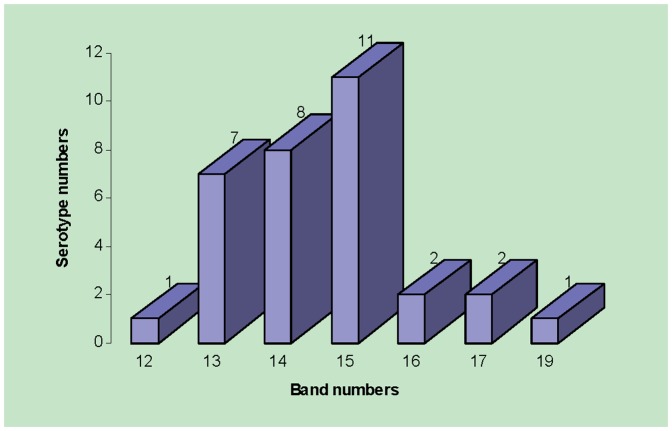
Band numbers of various *Salmonella* serotypes in the database. The number under each bar indicates the number of the bands, and the number on top of each bar shows the number of serotypes.

The normalized band matching of 45,923 PFGE patterns were displayed in one Excel file (data not shown). A total of 60 band locations were identified, ranging from 20 to 1100 kb for all the patterns in the database. The number of bands for each pattern and the overall mean of band numbers for each serotype were calculated (Figure 1). In the range of 20 to 1100 kb, most of the 32 serotypes (∼81%) had 13 to 15 bands; serotype Enteritidis had 12 bands; Paratyphi A had 19 bands; serotypes Heidelberg and Typhimurium var. 5- had 16 bands; and Typhi and Stanley had 17 bands.

**Figure 2:**
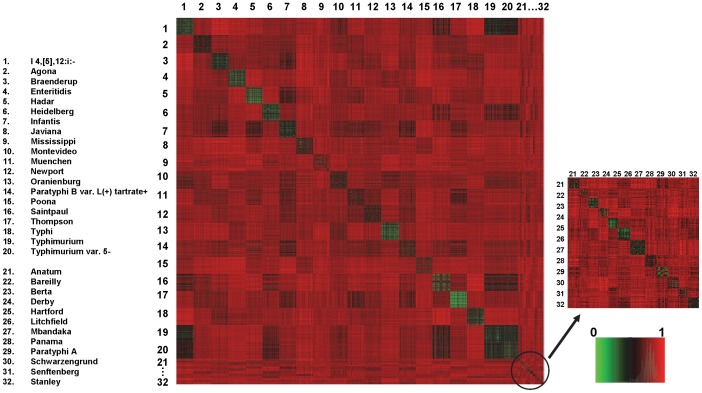
Distance matrix of 32 serotypes. The heatmap shows the distances matrix presenting the dissimilarities for any two patterns in the entire database. The dissimilarity of PFGE patterns inter- or intra-serotypes was calculated by Jaccard Distance, and the values ranged from 0 (green) to 1 (red) (shown in the index).

**Figure 3:**
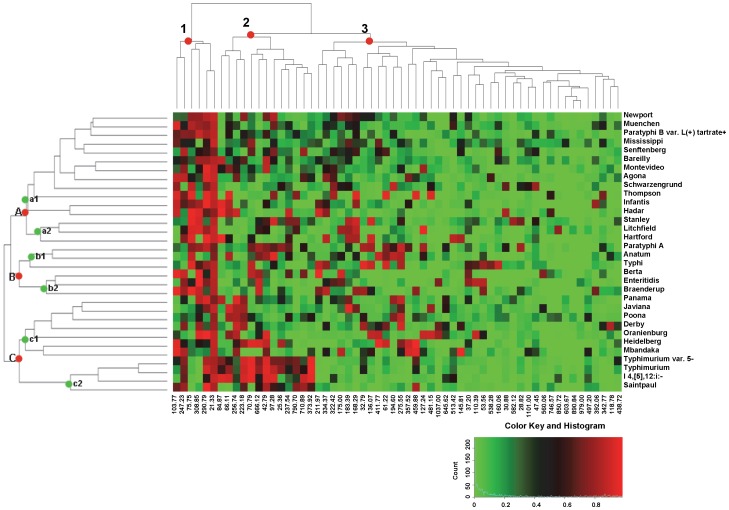
Two-way hierarchical clustering analysis of the 32 serotypes in the database. The color histogram shows the proportions of the bands present at every designated band location with values ranging from 0 to 1. The hierarchical cluster analysis was applied based on the dissimilarity measures of any two serotypes calculated by the Euclidean distance of the characteristic parameters. Both serotypes and band locations were clustered according to dissimilarity measures. The numbers 1, 2, and 3 and the letters A (a1, a2), B (b1, b2), and C (c1, c2) stand for the groups (and sub-groups) of band locations and serotypes.

For each serotype, the percentage of patterns containing bands of a particular size were determined to identify the most common bands associated with the patterns of the respective serotype. The 10 most frequent detected bands for each serotype were sorted and listed in Table 2 (for the 20 most common serotypes) and Table 3 (for the 12 less common serotypes). Within a particular serotype, the 10 most commonly detected bands were present in more than 50% of the analyzed patterns, with the exception of serotypes Mississippi (45%), Muenchen (49%) and Bareilly (49%). The top five bands were seen in more than 75% of the patterns for each of the serotypes, with the exception of the Mississippi (68%), Paratyphi B var. L(+) Tartrate+(74%), Poona (74%), and Senftenberg (68%). Several serotypes, including Hadar, Heidelberg, I 4, [5], 12:i:-, Thompson, Typhimurium, and Stanley, had more than 90% of the patterns containing the same top five bands and for serotypes I 4,[5],12:i:- and Thompson, 90% of the isolates shared the same top eight bands. The concordance of Tables 2 and 3 with Figures 2 and 3 is described in the Discussion.

**Table 2:** Top 10 most frequent bands and the percentages of 20 most frequent serotypes in the database.

Top 10 bands (kb)	1	2	3	4	5	6	7	8	9	10
Agona	290.79 (93%)	21.33 (89%)	103.77 (88%)	247.23 (86%)	334.37 (75%)	357.52 (73%)	256.7 (70%)	710.89 (65%)	322.42 (64%)	538.28 (63%)
Braenderup	103.77 (97%)	75.75 (97%)	666.12 (89%)	308.85 (89%)	334.37 (87%)	21.33 (85%)	247.2 (81%)	32.79 (81%)	168.29 (79%)	459.88 (77%)
Enteritidis	308.85 (98%)	666.12 (90%)	110.39 (86%)	53.56 (86%)	21.33 (86%)	247.23 (77%)	37.2 (77%)	1037 (74%)	290.79 (73%)	175.00 (70%)
Hadar	84.87 (98%)	75.75 (97%)	308.85 (93%)	211.97 (92%)	256.74 (90%)	237.54 (89%)	103.77 (89%)	30.88 (84%)	66.11 (83%)	334.37 (76%)
Heidelberg	459.88 (98%)	411.77 (98%)	103.77 (95%)	666.12 (94%)	70.79 (94%)	61.22 (90%)	357.52 (89%)	21.33 (89%)	42.79 (87%)	25.36 (83%)
I 4,[5],12:i:-	70.79 (96%)	373.92 (95%)	97.28 (94%)	84.87 (94%)	42.79 (93%)	223.18 (92%)	66.11 (92%)	710.89 (90%)	21.33 (89%)	247.23 (68%)
Infantis	290.79 (97%)	84.87 (95%)	103.77 (88%)	21.33 (87%)	66.11 (85%)	308.85 (81%)	392.06 (79%)	168.29 (78%)	75.75 (74%)	211.97 (68%)
Javiana	183.39 (95%)	66.11 (92%)	21.33 (88%)	256.74 (83%)	223.18 (81%)	481.15 (79%)	290.79 (74%)	168.29 (71%)	275.55 (68%)	645.62 (57%)
Mississippi	21.33 (89%)	70.79 (77%)	411.77 (70%)	103.77 (70%)	183.39 (68%)	290.79 (60%)	97.28 (48%)	342.77 (46%)	247.23 (46%)	75.75 (45%)
Montevideo	290.79 (95%)	21.33 (88%)	42.79 (87%)	127.24 (81%)	247.23 (77%)	66.11 (74%)	322.42 (63%)	256.74 (60%)	334.37 (56%)	308.85 (54%)
Muenchen	103.77 (93%)	21.33 (86%)	75.75 (85%)	308.85 (79%)	290.79 (76%)	97.28 (70%)	513.42 (63%)	66.11 (54%)	168.29 (53%)	247.23 (49%)
Newport	21.33 (89%)	290.79 (85%)	97.28 (85%)	308.85 (78%)	75.75 (78%)	223.18 (76%)	175.00 (73%)	42.79 (71%)	183.39 (67%)	168.29 (61%)
Oranienburg	290.79 (98%)	308.85 (95%)	42.79 (94%)	481.15 (90%)	127.24 (89%)	110.39 (89%)	21.33 (89%)	136.07 (88%)	411.77 (86%)	256.74 (85%)
Paratyphi B var. L(+)Tartrate+	308.85 (89%)	21.33 (87%)	290.79 (81%)	247.23 (75%)	75.75 (74%)	103.77 (68%)	42.79 (62%)	66.11 (56%)	168.29 (55%)	194.6 (52%)
Poona	275.55 (88%)	256.74 (83%)	66.11 (81%)	21.33 (78%)	223.18 (74%)	194.6 (74%)	308.85 (59%)	237.54 (59%)	32.79 (59%)	373.92 (54%)
Saintpaul	290.79 (98%)	21.33 (84%)	459.88 (83%)	42.79 (82%)	710.89 (80%)	247.23 (79%)	790.7 (75%)	103.77 (69%)	223.18 (67%)	373.92 (64%)
Thompson	75.75 (97%)	459.88 (95%)	290.79 (95%)	168.29 (95%)	66.11 (95%)	61.22 (95%)	481.15 (94%)	103.77 (93%)	21.33 (89%)	322.42 (82%)
Typhi	75.75 (95%)	32.79 (95%)	61.22 (92%)	160.06 (89%)	136.07 (89%)	211.97 (88%)	70.79 (87%)	21.33 (85%)	275.55 (83%)	53.56 (79%)
Typhimurium	373.92 (97%)	70.79 (97%)	42.79 (94%)	290.79 (92%)	223.18 (90%)	97.28 (87%)	21.33 (84%)	666.12 (78%)	66.11 (78%)	25.36 (75%)
Typhimurium var. 5-	373.92 (97%)	70.79 (96%)	42.79 (93%)	223.18 (91%)	290.79 (84%)	21.33 (83%)	247.23 (74%)	84.87 (73%)	97.28 (72%)	25.36 (72%)

**Table 3:** Top 10 most frequent bands and the percentages of 12 less frequent serotypes in the database.

Top 10 bands (kb)	1	2	3	4	5	6	7	8	9	10
Anatum	97.28 (95%)	411.77 (90%)	75.75 (88%)	194.6 (87%)	275.55 (84%)	66.11 (84%)	42.79 (80%)	21.33 (73%)	666.12 (72%)	61.22 (72%)
Bareilly	84.87 (87%)	42.79 (87%)	21.33 (85%)	290.79 (80%)	308.85 (79%)	223.18 (67%)	247.23 (63%)	97.28 (54%)	256.74 (51%)	160.06 (49%)
Berta	211.97 (98%)	308.85 (97%)	103.77 (96%)	175.00 (91%)	247.23 (89%)	21.33 (87%)	70.79 (86%)	666.12 (81%)	560.06 (74%)	710.89 (72%)
Derby	275.55 (91%)	256.74 (89%)	118.78 (84%)	223.18 (81%)	32.79 (81%)	308.85 (80%)	21.33 (80%)	247.23 (74%)	61.22 (71%)	1037 (69%)
Hartford	290.79 (98%)	84.87 (98%)	75.75 (94%)	136.07 (90%)	21.33 (89%)	513.42 (87%)	334.37 (86%)	145.81 (83%)	183.39 (82%)	168.29 (82%)
Litchfield	127.24 (97%)	308.85 (93%)	75.75 (93%)	21.33 (89%)	168.29 (86%)	175.00 (81%)	290.79 (79%)	183.39 (79%)	850.72 (77%)	790.7 (64%)
Mbandaka	145.81 (98%)	103.77 (95%)	459.88 (94%)	357.52 (92%)	175.00 (87%)	21.33 (87%)	247.23 (84%)	256.74 (77%)	42.79 (71%)	136.07 (60%)
Panama	256.74 (94%)	290.79 (93%)	275.55 (88%)	308.85 (86%)	84.87 (85%)	194.6 (78%)	223.18 (76%)	183.39 (72%)	21.33 (71%)	645.62 (59%)
Paratyphi A	275.55 (89%)	290.79 (87%)	32.79 (83%)	127.24 (82%)	666.12 (81%)	136.07 (81%)	28.82 (81%)	308.85 (79%)	25.36 (76%)	75.75 (75%)
Schwarzengrund	194.6 (88%)	75.75 (87%)	21.33 (86%)	47.45 (80%)	103.77 (77%)	322.42 (75%)	290.79 (72%)	30.88 (68%)	1101 (56%)	183.39 (56%)
Senftenberg	247.23 (85%)	710.89 (80%)	97.28 (73%)	21.33 (72%)	70.79 (68%)	160.06 (65%)	61.22 (65%)	168.29 (63%)	290.79 (62%)	322.42 (58%)
Stanley	75.75 (97%)	136.07 (93%)	308.85 (92%)	97.28 (92%)	168.29 (91%)	21.33 (87%)	183.39 (85%)	582.12 (81%)	790.7 (73%)	237.54 (73%)

### Distance matrix of the 32 serotypes

The heatmap of the distance matrix of 45,923 PFGE patterns for the 32 serotypes in the database is shown in Figure 2. Distances of the patterns were shown by large squares for the 20 most frequent serotypes, consisting of approximately 2,000 PFGE patterns for each serotype, and by small squares for patterns of the 12 less frequent serotypes, with approximately 200 to 500 PFGE patterns for each. The squares in the diagonal show the distances of the various patterns within the same serotype, while the other squares show the distances between the patterns of their corresponding horizontal and vertical serotypes. The squares on the diagonal of all 32 serotypes were distinguishable from other squares in the heatmap, except for the Typhimurium and Typhimurium var. 5-, and less distinguishable in I 4,[5],12:i:-. According to the CDC's annual report, [2], I 4,[5],12:i:- is the monophasic variant of Typhimurium (formula I 4,[5],12:i:1,2) and lacks the second phase H antigen 1,2. In surveillance reports, Typhimurium var. 5- has been considered an O:5-negative variant of Typhimurium or reported as Typhimurium [2]. No genetic differences were detected between these two variants [4]. Therefore, results of our study indicated that 29 out of 32 serotypes in the constructed database had patterns distinguishable from the others. The patterns for Typhimurium, Typhimurium var. 5-, and I 4,[5],12:i:- were similar and it was difficult to distinguish any differences.

The squares on the diagonal show various colors from green to black (Figure 2), indicating various degrees of similarities of patterns within the same serotypes. The bright green square of serotype Thompson indicated that 2045 patterns of this serotype were similar to each other; while the pale black square of serotype Mississippi shows that 1999 patterns of Mississippi in the database were distinctly different from each other, although relatively more similar compared to patterns in other serotypes.

### Hierarchical cluster analysis

To further characterize the PFGE patterns in the database, hierarchical cluster analysis was applied to the dissimilarity measures of any pair of serotypes calculated by the Euclidean distance of the characteristic parameters. At each column of designated band location, the color of each of the squares from green to red represented the various proportions (between 0 and 1) of band occurrences for each of the 32 serotypes. Figure 3 shows the result of two-way clustering analysis where both serotypes and band locations were grouped to identify the associations between serotypes and bands simultaneously. The 32 serotypes could be divided into 3 major groups (A, B, and C) with subgroups based on the dissimilarity measures of patterns of serotypes. Group A consisted of 15 serotypes (9 most frequent serotypes and 6 less frequent serotypes) and was sub-grouped into a1 and a2; Group B was composed of 6 serotypes (3 most frequent serotypes and 3 less frequent serotypes) and was sub-grouped into b1 and b2; and Group C had 11 serotypes (9 most frequent serotypes and 2 less frequent serotypes) and was sub-grouped into c1 and c2.

The 60 bands generated by band matching with BioNumerics software were divided into 3 major groups (Figure 3). Group 1 consisted of 6 bands with multiple red squares in the rows, indicating that these bands were commonly shared by the majority of patterns and serotypes. These bands are also listed in Tables 2 and 3 among the top 10 bands for various serotypes. Band 21.33 kb is a typical example. It appeared in all 32 serotypes at percentages from 70% to 89%, and was one of the top 10 bands for 30 of the 32 serotypes, the exceptions being Hadar and Paratyphi A. Group 2 was composed of 13 bands, which had fewer red squares in the rows and were distributed in fewer serotypes than group 1. These bands are also found in Tables 2 and 3 among the top 10 bands of the serotypes. For example, the column for band 373.92 kb has three bright red squares for serotypes Typhimurium var. 5- (0.97), Typhimurium (0.97), and I 4,[5],12:i:- (0.95), and one dark red square for serotype Saintpaul (0.64) (Figure 3). The rest of the squares in this column are all green, indicating that the rest of the serotypes had low percentages at this location. This band was ranked as the top band for both serotypes Typhimurium and Typhimurium var. 5-, as the 2^nd^ most frequent band for serotype I 4,[5],12:i:-; and as the 10^th^ most frequent band for serotype Saintpaul (Table 2). The rest of the 41 bands belonged to group 3. In this group, the red squares are fewer and distributed more sporadically among the serotypes than in groups 1 and 2. This group could be further divided into several sub-groups. The bright red squares are distributed more in the left half of the bands and only a few in the right half. For example, at location 118.78 kb, 84% of the patterns of serotype Derby showed bands, and this band was ranked as the 3^rd^ most frequent (Table 3). Limited patterns of fewer serotypes show bands at locations of 603.67, 890.84, 979.00, 497.20, and 438.72 kb in the right sub-groups of Figure 3, and the proportions were less than 50%.

## Discussion

Since PulseNet has established a standardized PFGE protocol and an extensive quality assurance system to enhance data comparability and interpretation [27], PFGE results have high reproducibility between laboratories following the standard protocol and guidance from CDC. The present work included as many frequently occurring serotypes and PFGE profiles as possible in our database to reflect the trends identified in *Salmonella* surveillance programs in the US during the years 1996−2009. The database should make it easier to systematically evaluate the performance of PFGE for subtype discrimination, and to enable more accurate meta-analyses based on a sufficient data set size. Our research group has applied this database on developing a system for rapid prediction of *Salmonella* serotypes based on the PFGE fingerprints [28], [32].

Although PFGE has been applied extensively in the epidemiological investigation and surveillance of *Salmonella* for the last two decades, only a few systematic investigations have been pursued on the phylogenetic relationships among PFGE patterns and *Salmonella* serotypes [6], [28], . Liebana et al. compared several methods for discriminating *Salmonella* isolates of five serovars, inferring that certain serotypes could be deduced solely by their PFGE patterns [33]. The correlation of serotypes to PFGE patterns was further described by Gaul *et al.* [34] based on an analysis of 674 isolates from 12 *Salmonella* serotypes, concluding that PFGE fingerprints could potentially provide an alternative method for screening and identifying *Salmonella* serotypes. In 2007, Kerouanton et al. set up a database of 1128 PFGE patterns of 31 *Salmonella* serotypes, and evaluated the subtype discrimination of the PFGE method according to the standard PulseNet protocol [6]. Cluster analysis was used on the PFGE patterns to confirm that serotype and PFGE genotype were closely linked in the three studies. The serotypes and number of PFGE patterns included in these studies were limited. In this work, we applied the meta-analysis on the PFGE patterns in the large database, and used bioinformatics methods to identify both the inter- and intra-serotypes relationships of 32 frequently occurring serotypes for the first time.

*Salmonella* serotypes can be closely related in terms of virulence, prevalence and antimicrobial resistance [12], [13], [26], [35], [36] and PFGE has been successfully used for the characterization of several serotypes [6], [28]. However, the discrimination of PFGE varies with serotype [28], [33]. The meta-analysis of the band numbers of the 32 serotypes in our database revealed that Enteritidis and Paratyphi A had unique band numbers (Figure 1). In the range of 20 to 1100 kb, most of the 32 serotypes had 13 to 15 bands on the average, while only Enteritidis had 12 bands and only Paratyphi A had 19 bands on the average (Figure 1). These deviating band numbers could be used as a marker to distinguish these two serotypes from the other 30.

This study is the first report providing comprehensive summary of the 10 most frequent bands present in each serotype and their occurrence for each of the 32 most frequent serotypes (Tables 2 and 3). Most of the bands showed up in more than one serotype, but with different frequencies and rank orders. This result could be visually confirmed by hierarchical clustering analysis of the dissimilarity measures of any two serotypes, calculated by the Euclidean distance of the characteristic parameters (Figure 3). The six bands of group 1 in Figure 3 were shared by most of the 32 serotypes as the top 10 bands (Tables 2 and 3), especially the band of 21.33 kb. It was the only band that was shared by high percentages of patterns in all of the 32 serotypes. The other five bands in group 1, although well distributed in most of the patterns of most of the serotypes, were absent from some of the serotypes (shown as bright green squares, Figure 3). For example, four serotypes in group c2 (Figure 3) lacked bands at 75.75 kb and 308.85 kb, which differentiated these serotypes from the others. The bands in groups 2 and 3 (Figure 3) seemed to be more serotype-specific. In particular, the bands in the right half of group 3 showed uniqueness for serotypes, which may possibly be used as marker bands to rapidly distinguish serotypes. Since the characteristic parameters of serotype dissimilarity originated from the 45,923 patterns in the database (∼2000 patterns for the 20 most frequent serotypes and ∼200−500 for the 12 less frequent serotypes), the image of each row in Figure 3, including the band location and frequency, could be considered the reference fingerprint for each serotype. Considering that PFGE fingerprints were obtained from various laboratories and slight variations may occur when band matching of BioNumerics software is applied to different combinations of PFGE patterns, the band sizes may vary to a small extent.

The distance matrix (Figure 2) presented the similarities/dissimilarities of any two patterns in the database. Figure 3 illustrated further the groups of the serotypes derived from the dissimilarity measures of any of two serotypes, based on the distance calculations of the serotype characteristic parameters. These results were concordant with each other and with the results shown in Tables 2 and 3. For example, serotypes Typhimurium, Typhimurium var. 5-, I 4,[5],12:i:-, and Saintpaul were grouped in group c2 (Figure 3) because they demonstrated less dissimilarity with each other than with the other serotypes. These four serotypes shared five to seven of their top 10 most frequent bands (Tables 2 and 3), and showed the close distances in black to dark green squares, corresponding to the four serotypes, horizontally and vertically (Figure 2). I 4,[5], 12:i:- lacks the second phase H antigen 1, 2 and is the monophasic variant of serotype Typhimurium due to antigenic and genotypic similarities between the two serovars [37], [38]. The serotype Typhimurium var. 5-, whose previous name was Typhimurium var. Copenhagen, was considered as an O:5-negative variant of serotype Typhimurium a few years ago [2], [4]. The close relationships and similar PFGE patterns of Saintpaul to Typhimurium, Typhimurium var. 5-, and I 4,[5],12:i:- was concordant with that of Didelot *et al*. [39], in which 12 strains of Typhimurium and Saintpaul were grouped in the same ancestral population by applying the linkage model on enhanced MLST sequencing data. Based on the results in the current study, we first reported the close relationship of PFGE patterns between serotypes Hadar to Infantis and Muenchen to Newport (Figures 2 and 3, Tables 2 and 3). The high percentages of the top 10 bands (Tables 2 and 3) resulted from the high similarities among the patterns within the particular serotypes (green squares diagonally displayed in Figure 2). For example, isolates of I 4,[5],12:i:- and Thompson each had 90% of their isolates harboring the same top eight bands. However, Mississippi showed relatively lower proportions for the top 10 bands, reflected as the pale black square in the heatmap of the distance matrix (Figure 2).

This study has highlighted the use of meta-analysis on the constructed large database of 45,923 PFGE profiles of 32 *Salmonella* serotypes from human sources. The constructed database provided a platform to study the relationships between phenotypes and genotypes of *Salmonella* isolates. From our data, we conclude that certain serotypes have higher degrees of diversities of their PFGE patterns compared with the majority of other serotypes. The results of the meta-analysis indicated that the pattern similarities/dissimilarities determined the serotype discrimination of PFGE method, and that the possible PFGE markers may have utility for serotype identification. The presence of distinct, serotype specific patterns may provide useful information to aid in the distribution of serotypes in the population and potentially reduce the need for laborious analyses, such as traditional serotyping. Future studies combined with the *Salmonella* genome sequencing data will be critical to match PFGE patterns to NGS data. The connection between PFGE ‘gold standard’ and the new NGS technology will be very helpful for PFGE data retrieval and interpretation, and will greatly improve and accelerate the rapid detection and identification of pathogens and source tracking in the “-omics” era.
